# Multi-View Data Integration Methods for Radiotherapy Structure Name Standardization

**DOI:** 10.3390/cancers13081796

**Published:** 2021-04-09

**Authors:** Khajamoinuddin Syed, William C. Sleeman, Michael Hagan, Jatinder Palta, Rishabh Kapoor, Preetam Ghosh

**Affiliations:** 1 Department of Computer Science, Virginia Commonwealth University, Richmond, VA 23284, USA; william.sleemaniv@vcuhealth.org (W.C.S.IV); pghosh@vcu.edu (P.G.); 2 Department of Radiation Oncology, Virginia Commonwealth University, Richmond, VA 23298, USA; michael.hagan@vcuhealth.org (M.H.); jatinder.palta@vcuhealth.org (J.P.); rishabh.kapoor@vcuhealth.org (R.K.); 3 National Radiation Oncology Program, Department of Veteran Affairs, Richmond, VA 23249, USA

**Keywords:** radiotherapy structure names, weighting techniques, multi-view data integration, machine learning, image classification, text categorization, TG-263

## Abstract

**Simple Summary:**

Structure names associated with radiotherapy treatments need standardization to develop data pipelines enabling personalized treatment plans. Automatic classification of structure names based on the currently available TG-263 nomenclature can help with data aggregation from both retrospective and future data sources. The aim of our proposed machine learning-based data integration methods is to achieve highly accurate structure name classification to automate the data aggregation process. Our multi-view models can overcome the challenges of integrating different data types associated with radiotherapy structures, such as the physician-given text labels and geometric or image data. The models exhibited high accuracy when tested on multi-center and multi-institutional lung and prostate cancer patients data and outperformed the models built on any single data type. This highlights the importance of combining different types of data in building generalizable models for structure name standardization.

**Abstract:**

Standardization of radiotherapy structure names is essential for developing data-driven personalized radiotherapy treatment plans. Different types of data are associated with radiotherapy structures, such as the physician-given text labels, geometric (image) data, and Dose-Volume Histograms (DVH). Prior work on structure name standardization used just one type of data. We present novel approaches to integrate complementary types (views) of structure data to build better-performing machine learning models. We present two methods, namely (a) intermediate integration and (b) late integration, to combine physician-given textual structure name features and geometric information of structures. The dataset consisted of 709 prostate cancer and 752 lung cancer patients across 40 radiotherapy centers administered by the U.S. Veterans Health Administration (VA) and the Department of Radiation Oncology, Virginia Commonwealth University (VCU). We used randomly selected data from 30 centers for training and ten centers for testing. We also used the VCU data for testing. We observed that the intermediate integration approach outperformed the models with a single view of the dataset, while late integration showed comparable performance with single-view results. Thus, we demonstrate that combining different views (types of data) helps build better models for structure name standardization to enable big data analytics in radiation oncology.

## 1. Introduction

An important aspect of radiation therapy is to reduce the exposure of radiation to healthy tissue while delivering enough dose to the cancer. During the treatment planning process, the radiation oncologist identifies different regions (structures) of the body and labels them on Magnetic Resonance Imaging (MRI) or Computed Tomography (CT) scans. The delineated structures are categorized as Organs-At-Risk (OARs),targets (PTV (Planning Target Volume), GTV (Gross Target Volume, CTV (Clinical Target Volume)), and other (planning-related structures). The textual labels for these structures are often inconsistent because of individual physician preferences, the properties of the treatment-planning systems, and different policies between radiotherapy clinics. These inconsistencies make it difficult to aggregate retrospective or future data sources.

Table 1 includes an example of the inconsistencies often found in the naming practices of radiotherapy structures. It shows that even though the patients come from the same institution, the names used to represent the same structures can be completely different. This issue can be compounded when these data come from multiple institutions.

Standard nomenclatures are imperative for constructing population-scale datasets. They enable automatic data extraction from electronic medical records for creating informatics pipelines in support of designing clinical trials, Quality Assurance (QA) of clinical and treatment processes, and ultimately improving clinical practices [1,2]. However, adhering to a standardized nomenclature can only address future data issues; this does not resolve inconsistencies in retrospective data. One solution is to relabel the structure names with standardized names manually. However, manual relabeling is not feasible for data coming from multiple centers because it is both inefficient and non-scalable, resulting in considerable investments of time and effort. To address these limitations of retrospective structure name standardization, we propose automated machine learning-based multi-view data integration approaches.

Our contributions: In this paper, we present a novel methodology for structure name standardization by combining textual physician-given names and geometric information (from imaging datasets). The specific contributions are the following:

1. We demonstrate that the combination of the textual features (physician-given structure names) and image features (geometric information structures) helps in improving the structure name standardization process.
2. We show that even the PTV structure can be identified along with the OARs with the physician-given names.
3. We demonstrate that it is still challenging to predict the standard name with only geometric information in real-world clinical datasets.
4. We demonstrate that late integration (combining at the prediction level) reduces the false positives and intermediate integration (combining at the feature level) performs better overall on metrics than single-view models on multi-center radiotherapy datasets.

## 2. Related Work

In our previous work [3], we identified three main categories for standardizing structure names, namely ontology-based, machine learning-based, and expert-based [4]. In this section, we focus on only the machine learning approaches, as this is the category of our proposed methodology.

One such approach used text-based features for standardization by generating feature vectors using different types of measures for string similarity; next, a classification algorithm was used to predict labels based on these feature vectors [5]. The authors used neural networks for classification; however, they did not provide enough details on the model, which are necessary to reproduce their results.

In our previous work on text-based classification [3], physician-given textual names were used to generate word embedding features, and the supervised fastText algorithm was then used to build a disease-specific structure name standardization model. It was demonstrated that such names given by physicians contain relevant information to predict the standard name of structures.

Geometric information was also used in prior works [6,7] for standardizing structure names. These works leveraged neural networks for structure name standardization considering the head and neck anatomical region. Although good accuracy was reported, they ignored the Non_OAR structures while also considering a limited number of OARs in their model. However, removal of Non_OAR structures makes these methods impractical for real clinical datasets as Non_OAR structures are usually present and often are the most prevalent structure type.

We also presented a different structure name standardization approach using the geometric information of structures [8]. By including bony anatomy data and additional information about the other structures for the same patient, we demonstrated that traditional machine learning methods such as random forests could achieve similar classifier accuracies as deep neural networks when only considering OAR structures. In addition, we also illustrated that including both OAR and Non_OAR structures makes the structure name relabeling problem much more difficult.

## 3. Materials and Methods

### 3.1. Dataset

The U.S. Veterans Health Administration (VA) has 40 radiation therapy centers distributed nationally. A major clinical informatics initiative from the VA is the implementation of the Radiation Oncology Quality Surveillance Program (VA-ROQS) [9] to assess the quality of treatments across these national centers. In the first phase of the VA-ROQS program, clinical data were physically retrieved from these 40 different centers from a variety of informatics platforms including clinical charts stored in the electronic medical records, DICOM (Digital Imaging and Communication in Medicine) files from imaging platforms, and other proprietary clinical systems for radiation therapy such as the treatment management and planning systems. A maximum of 20 patients were considered for each of the prostate and lung cancer categories for every VA center following the selection criteria delineated in [9], which resulted in aggregating clinical data for 709 prostate and 752 lung cancer patients. In practice, both CT and MRI scans are used for treatment planning. However, we used only the CT image dataset in this work. This dataset was comprised of the textual physician-given structure names [3], as well as the DICOM CT imaging data [8], among other fields. Physicians also performed manual labeling of seven prostate and five lung OAR structures using the TG-263 nomenclature, while the remaining structures were classified as other.

Additionally, we prepared a separate dataset comprised of DICOM CT imaging data from a random cohort of 50 prostate cancer patients and also 50 lung cancer patients; this dataset was compiled from the Radiation Oncology department at Virginia Commonwealth University (VCU). A similar process was used for manually labeling the structures in this VCU dataset. The dataset from the VA radiotherapy treatment centers is referred to as the VA-ROQS dataset, and the dataset from the VCU’s radiation oncology department is referred to as the VCU dataset in the rest of this paper. The prostate and lung structures (OARs and PTV) considered in this work are listed below.

Prostate structures (OARs and PTV): Femur_L, Femur_R, Bowel_Large, Bowel_Small, Bladder, Rectum, PTV.

Lung structures (OARs and PTV): Esophagus, SpinalCord, Brachial_Plexus, Heart, PTV.

Table 2 shows the distribution of prostate and lung structures for the VA-ROQS and VCU datasets. It is clear from the table that structures labeled as “others” are the highest occurring structures. The “other” category includes all structures contoured during treatment planning, delivery, and dose evaluation. We further noticed that imbalance in the OARs, PTV, and other categories was consistent in each center. Table 1 shows the examples of physician-given names across different patients for the same OARs, PTV, and others structures, and it also highlights the variability in the physician-given names. Finally, Table 2 also shows the number of unique physician-given names found in the VA-ROQS and VCU datasets for each lung and prostate structure.

As mentioned earlier, the data used in this work were initially collected for another project, which concentrated on overall treatment quality assessment [9]. The prostate and lung disease sites were chosen as they covered most of the patients receiving radiotherapy each year at the VA radiotherapy treatment centers. In addition, to the best of our knowledge, this may be one of the biggest (if not the biggest) datasets that includes full radiotherapy treatment DICOM data with annotated OARs/PTV structures. We chose to use this dataset because of the clinical relevance and the relatively large number of patients. Going forward, we are interested in looking at the smaller available datasets for other disease sites and trying transfer learning-based approaches from the larger prostate or lung models; however, this is out of scope of the current paper.

### 3.2. Creation of the Structure Set

One of the first steps in radiotherapy is to identify the anatomical regions of interest that should be irradiated or avoided during treatment. After the patient is imaged, often with CT or MRI, the physician uses a Treatment Planning System (TPS) to draw a border around each of these regions, also called structures. In this work, we only considered CT imaging data. This process was repeated for all relevant imaging slices until the delineation was complete, which resulted in a series of closed polygons for each individual structure. All of the structure data created for a specific patient were stored as a structure set file in the DICOM format, which is the current domain standard for storing and communicating medical imaging and radiotherapy data.

### 3.3. Data Preprocessing

#### 3.3.1. Textual Data Preparation

The maximum length and character set allowed for use in naming the radiotherapy structures are vendor dependent. Hence, in our dataset, we noticed that even with the high variability in structure naming practices, the overall character set used for naming was limited. Table 1 shows structure names’ variability across different patients from the VA-ROQS dataset. Text-based preprocessing techniques need to be carefully chosen in such cases where important details cannot be pruned, which may lead to lower accuracy in standardization. Therefore, we applied minimal preprocessing to the names given by physicians and simply converted them to lowercase.

#### 3.3.2. Geometric Data Preparation

The geometric-based feature vector was created from the DICOM planning image and structure set based on our previous work [8]. The planning image was used to create the clinical treatment plan, which included delineating the anatomical regions of interest. The delineation process was performed by either manually outlining each structure per image slice or with an automatic or semi-automatic tool that was part of the treatment planning system. These delineations were stored as DICOM structure sets in which they were represented by a series of 2D points per image slice.

To convert these data into a feature vector, the bounding box of the planning image was first calculated. Since the structure set and image bounding box belong to the same coordinate system, the structure set points can be interpolated onto the image grid. For each image slice, the consecutive interpolated points were connected with line segments to form closed polygons, and the polygons for each anatomical region were then filled to create a solid volume. The final feature vector was created by converting the 96 × 96 × 48 bitmap and converted to a 1D vector of length 442,368.

In addition, the bony anatomy information from the planning image was also extracted, thereby adding context to the locations of the delineated regions. The planning image, represented as Hounsfield Units (HU), was subjected to a threshold, so values above 1300 HU were converted to the value of 1, and all values below were set to 0. As with the structure set data, this volume was converted to a vector of length 442,368.

Experiments were performed with just the structure set feature vector and the concatenation of the structure and bony anatomy data. Since either combination of geometric data resulted in very long feature vectors and may lead to the curse of dimensionality [10], we performed feature reduction using truncated Singular-Value Decomposition (SVD) down to different total features. We tested the top 50, 100, 250, 500, and 1000 features. These steps were discussed comprehensively in [8].

### 3.4. Multi-View Data Integration Methods

Multi-view integration involves joining distinct feature sets of possibly different modalities to get the global view of the data. Depending on the data types, multi-view integration methods can be divided into three main categories based on when the heterogeneous data are integrated.

- **Early integration**: In this method, data from different views were concatenated to form a single feature space. Our dataset was heterogeneous (text and image) in nature, and hence, simple concatenation was not a feasible solution. Each structure was 442,368 features long (considering the imaging features) when it was converted to a single-dimensional binary vector. Simple concatenation of the image feature vector to other features was not feasible here because an increase in dimensionality negatively affected the model training and performance. For that reason, we decided to not implement this approach of integrating the features.
- **Intermediate integration**: This method converted all the data sources into a common or reduced feature space. This is also known as the transformation-based integration method. Next, the reduced feature vectors were concatenated to construct the final feature vector. The final vector was used to train and optimize the final model.
- **Late integration**: This method used data from each view to be separately analyzed, and then, the results from each view were integrated. This method has two main advantages over other integration types for heterogeneous data. First, the best suitable algorithm can be chosen depending on each data type. Second, each model is independent of each other and has the opportunity to be executed individually.

In this work, we only implemented the intermediate and late integration approaches. Details of these two approaches are described in Section 3.5.

### 3.5. Model Selection

#### 3.5.1. Single View

The dataset used in this work was heterogeneous in nature. To properly compare the advantages of utilizing the multi-view heterogeneous data, we built the best possible models with a single view separately. In our previous work, we thoroughly investigated the different algorithms for standardizing radiotherapy structure names with physician-given names [3] and geometric information [8] separately. The single-view model selection details are summarized below.

- Text data (physician-given structure names): We built the structure name standardization models using the combination of different feature extraction techniques, feature weighting, and ML algorithms. We tested NGram(unigram, bi-gram, and tri-grams), character n-grams, and word embedding techniques for feature extraction. For feature weighting, we evaluated the term presence (tp), term count (tc), term frequency (tf), and term frequency-inverse document frequency (tf-idf) techniques. Finally, we compared the following six ML-based classification methods to select the initial model: k-Nearest Neighbors (KNN) [11], SVM-linear [12], SVM-RBF [13], Random Forest (RF) [14], Logistic Regression (LR) [15], and fastText [16]. The scikit-learn library for machine learning [17] was used to build the models. Finally, we selected the fastText algorithm for automatically identifying the standard structure names using the physician-given names based on the performance comparison with other algorithms.
- Image data (3D geometric information of structures): In our initial work, we investigated the radiotherapy structure name standardization using geometric information. In order to extract geometric information, we converted the geometric information into binary vectors and selected the top 100 components with a truncated SVD algorithm. Having thoroughly tested different algorithms, we used the random forest classification algorithm to build our final model [8].

#### 3.5.2. Intermediate Integration

Intermediate integration involves transforming the multi-view data into a similar feature space and combining them (concatenating) into one. We utilized different techniques to transform them into a similar feature space as follows.

- Image data transformation: We used the image data transformation explained in Section 3.3.2. However, we used the first 50 principal components in this method, which produced better results.
- Text data transformation: We used the fastText algorithm to generate the word embeddings of physician-given names (numerical representation) of size 200.

**Figure 1:**
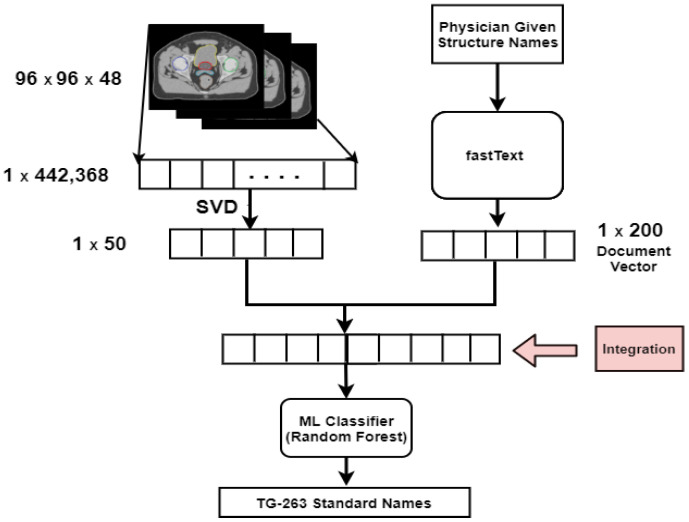
Intermediate integration method architecture for structure name standardization.

A final vector of size 250 was generated by concatenating feature vectors from each view. This vector was fed into the ML algorithm. We compared the four algorithms, RF, LR, and SVM with the linear and RBF kernel. We chose the RF algorithm to build a final intermediate integration model. Figure 1 shows the pictorial representation of intermediate integration.

#### 3.5.3. Late Integration

**Figure 2:**
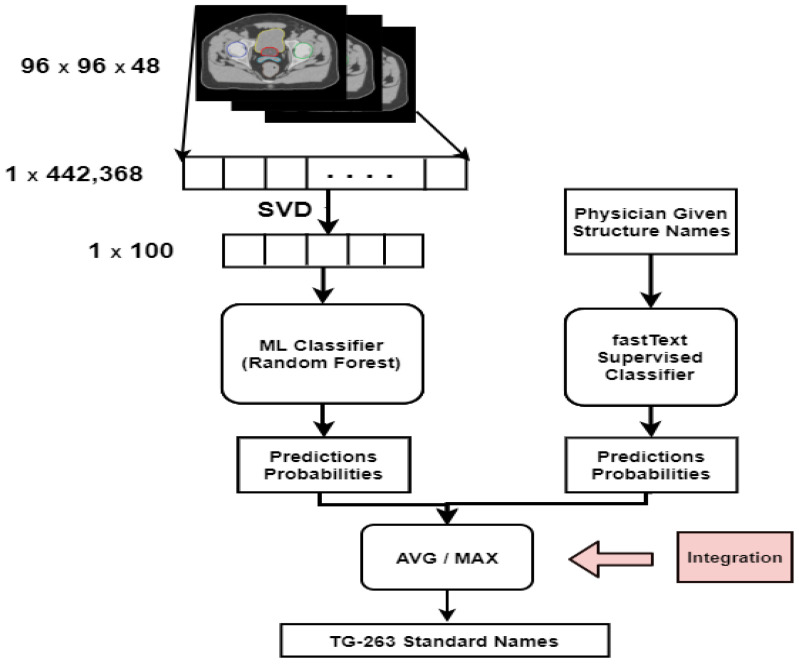
Late integration architecture for structure name standardization.

In late integration, separate models were built on each view, and the prediction probabilities from each model were then integrated to generate the final result. Figure 2 shows the pictorial representation of late integration. This is also known as model-based integration. Integrating at a late stage has an advantage over other types of integration; the best algorithms for each view (text and image) can be selected, and each model can run in parallel. We used a random forest algorithm for image features and a fastText algorithm for text features; a prediction probability vector was generated from each model instead of class prediction. The prediction probability vector’s length was equal to the number of classes in that dataset; it was eight for the prostate and six for the lung dataset. The resulting vector from each view was then combined to generate the final prediction probabilities. These final prediction probabilities were used to predict the final class prediction.

We used two techniques to combine the prediction probabilities from each view.

- Average (AVG): We created the final prediction probability vector by adding element-wise from each view and dividing it by the number of views. The final class was selected whose AVG probability was the highest.
- Maximum (MAX): We selected the maximum from each view, and the resulting vector contained the maximum for each class from all the views. The final class was predicted by selecting the class from this resultant vector with the highest probability.

### 3.6. Model Evaluation

The main objective of building any machine learning system is to demonstrate its quantifiable generalizability; to that end, a classification algorithm’s goal is to predict the correct label from the unseen samples. Using the same dataset for training and evaluation leads to an overestimation of the model’s performance. A hold-out dataset was used to get a more realistic model performance. Model validation was preferred using the full dataset instead of a single hold-out set [18,19]; one popular technique for this was k-fold cross-validation.

Hence, we randomly chose the 30 centers’ data from the VA-ROQS dataset for training and the remaining ten centers’ data for testing. Along with the VA-ROQS testing dataset, we also tested with the VCU dataset (external dataset).

We used the two techniques mentioned below to validate our models.

- K-fold cross-validation: We split the VA-ROQS data into *K*-folds so that the individual folds were stratified by their corresponding standard names. Next, *K*-1 folds were used for the training phase, while the other fold for validation. We continued this process in order to validate all of the folds. Here, *K* indicated the number of folds in which the data were divided, and we used 5 folds.
- VA center-based cross-validation: In this technique, the data from each center were validated separately. Data from 30 centers were further divided such that 2 (*n* − 1) centers were utilized for the training phase, while the other center’s data were used for validation; this was repeated till each of the centers were validated.

#### Model Testing

The final model was built after thorough validation and testing with unseen data. We tested our model with the two datasets below, which were not used during training and validation in this work.

- VA center-based test: We randomly selected 10 VA centers’ data used for testing. With this dataset, we were able to test the model’s ability to generalize on data from multiple VA centers.
- VCU test: We used the data from the VCU to test the model’s ability to generalize over the data coming from outside of the VA centers.

### 3.7. Performance Measures or Evaluation Metrics

The dataset used in this work was highly imbalanced. Hence, the metrics used to evaluate the models built with imbalanced data needed to be agnostic to the the data imbalance. Since the datasets were multi-class, we used the macro-averaged metrics instead of the micro-averaged ones. Macro-averaged metrics give equal importance to the classes regardless of the number of samples in a given class.

The formal expressions for the metrics are as follows.

(1) $$Precision_{macro}=\frac{1}{N}\sum_{c=1}^{N}\frac{TP_{c}}{TP_{c}+FP_{c}}$$

(2) $$Recall_{macro}=\frac{1}{N}\sum_{c=1}^{N}\frac{TP_{c}}{TP_{c}+FN_{c}}$$

(3) $$F_{1}score_{macro}=\frac{1}{N}\sum_{c=1}^{N}2\cdot \frac{Precision_{c}\cdot Recall_{c}}{Precision_{c}+Recall_{c}}$$

(4) $$Accuracy=\frac{TP+TN}{TP+TN+FP+FN}$$

Here, TP are the True Positives, TN are the True Negatives, FP are the False Positives, and FN are the False Negatives.

We also used the confusion matrices to assess the model performance. The number of predictions that were correct/incorrect were depicted by the count values and further divided for each class. The confusion matrix provided an insight into a model’s confusion between classes, which was important to understand the types of errors made by the model.

## 4. Results

Here, we discuss the performance of our proposed approaches. The results are divided into three subsections: singe-view results, intermediate integration, and late integration results. In order to provide a better comparison with the single-view, intermediate integration, and late integration results, we built a baseline model. Considering the highly imbalanced dataset at hand, we built a Majority Label Baseline (MLB) model. In this model, the model learns to predict the class that occurs the maximum number of times in a training dataset; here, the assumption was that the maximum number of occurring labels or class was of type “other”. When we calculated the classification metrics, we observed that the maximum F${}_{1}$-score achieved on all four datasets was below 0.15. Anything above this F${}_{1}$-score indicated that the model can learn patterns from the training data.

### 4.1. Single-View Results

**Figure 3:**
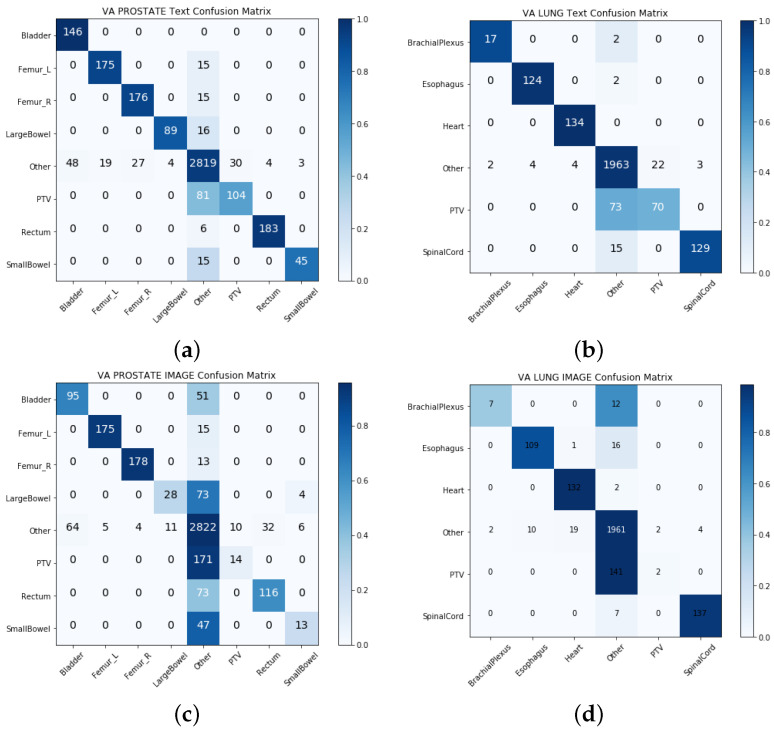
Single-view results: (**a**) VA-ROQS prostate text-based features. (**b**) VA-ROQS lung text-based features. (**c**) VCU prostate image features. (**d**) VCU lung image features. A darker color demonstrates more accurate prediction, and the diagonal shows the labels predicted correctly.

**Figure 4:**
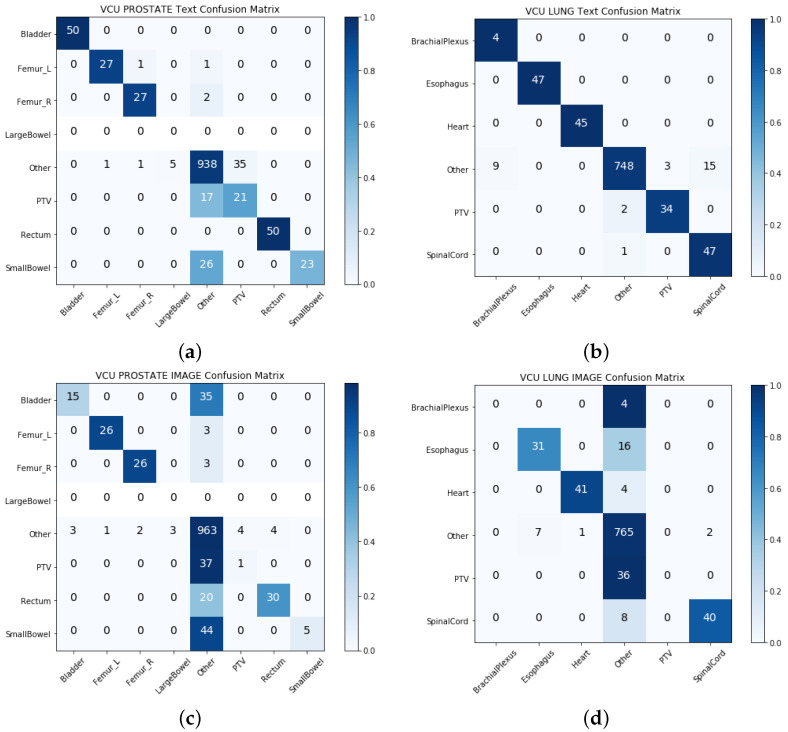
Single-view results: (**a**) VCU prostate text-based features. (**b**) VA-ROQS lung text-based features. (**c**) VCU prostate image features. (**d**) VCU lung image features. A darker color demonstrates more accurate prediction, and the diagonal shows the labels predicted correctly.

We built two separate models with physician-given structure names (textual data) and the geometric information of structures (geometric data) in the single-view approach. Table 3 and Table 4 show the model performance for the VA-ROQS and VCU datasets for both prostate and lung cancers. We observed that models utilizing the structure names consistently outperformed the models built utilizing geometric information. Geometric information-based models performed much better than the baseline model; however, they were the poorest performing models compared with the text-based models and other multi-view integrated models. We also noticed that the text-based model performed better than the intermediate integration model on lung structures from both datasets with an F${}_{1}$-score of 0.893 on the VA-ROQS lung dataset and 0.873 on the VCU lung dataset. We observed that the text-based model had an F${}_{1}$-score of 0.872 for the VA-ROQS prostate dataset and 0.74 for the VCU prostate dataset. Figure 3 and Figure 4 show the confusion matrix for both the VCU and VA-ROQS datasets. The VCU prostate dataset had no instances of “large bowel” structures in the dataset, but the model predicted “large bowel” for three structures. Tables S1–S4 in the Supplementary Materials show the comparative results for the VA-ROQS and VCU data for both diseases.

### 4.2. Intermediate Integration Test Results

**Figure 5:**
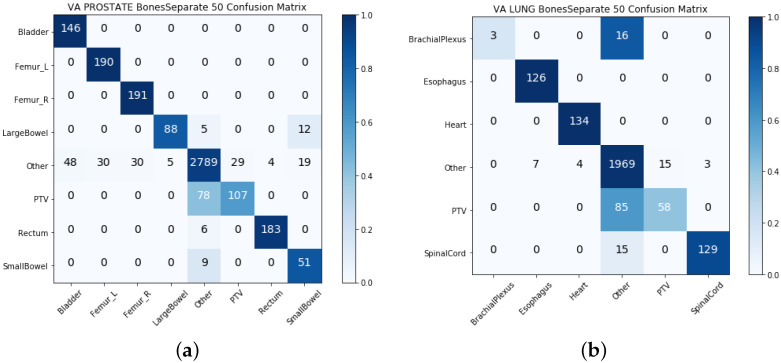

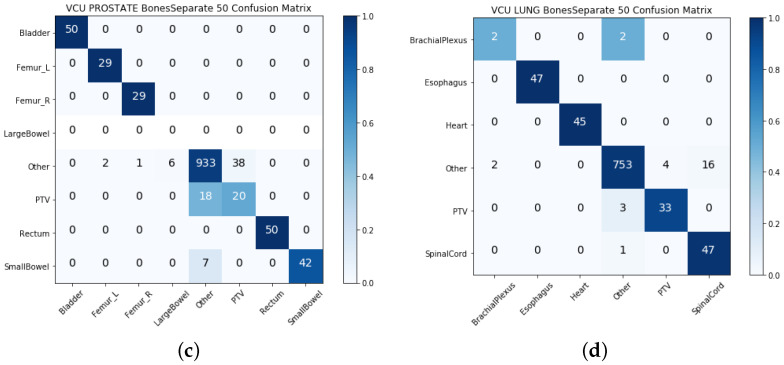
Intermediate integration: VA-ROAQS and VCU lung dataset confusion matrix. (**a**) Text-based features, (**b**) image features, (**c**) AVG of predictions, and (**d**) MAX of two predictions. A darker color demonstrates more accurate prediction, and the diagonal shows the labels predicted correctly.

In this method, we transformed the structure names and geometric information into similar feature spaces. These two features spaces from different views were then concatenated for ML model training. Tables S5–S8 show that the combination of the top 50 principal components and a document vector of size 200 with the random forest algorithm performed best on three out of four datasets. Hence, all the subsequent results reported in Table 3 were based on this combination, which show the macro-averaged precision, recall, and F${}_{1}$-score for intermediate integration models on all four datasets. We observed that the intermediate integration method performed better on prostate structures on both the VA-ROQS and VCU datasets. Precision was higher for the single-view models with three out of four datasets, while the overall F${}_{1}$-score was higher on both the VA-ROQS and VCU prostate datasets. The increase in precision indicated that the model predicted fewer false positives for the OAR and PTV structures. Figure 5 illustrates the confusion matrices for prostate and lung structures in the VA-ROQS and VCU datasets. Intermediate integration consistently reduced the false positives for all OAR and PTV structures and increased the false positives in the other structures. Tables S17–S20 in the Supplementary Materials show the intermediate integration models’ label-wise results on all four datasets.

### 4.3. Late Integration Test Results

**Figure 6:**
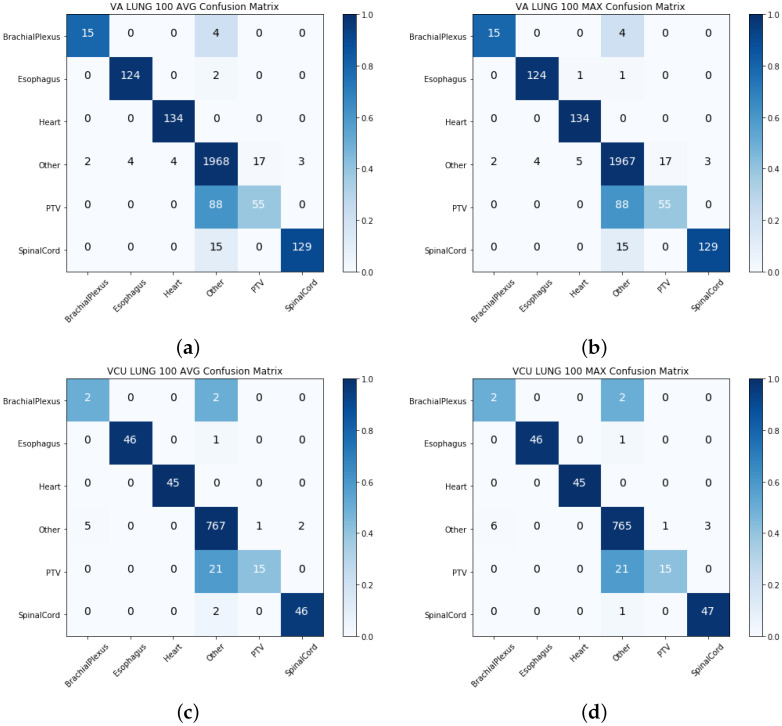
Late integration: confusion matrix for VA-ROQS and VCU lung datasets: (**a**) VA-ROQS lung AVG integration, (**b**) VA-ROQS lung MAX integration, (**c**) VCU lung AVG integration, and (**d**) VCU lung MAX integration. A darker color demonstrates more accurate prediction, and the diagonal shows the labels predicted correctly.

**Figure 7:**
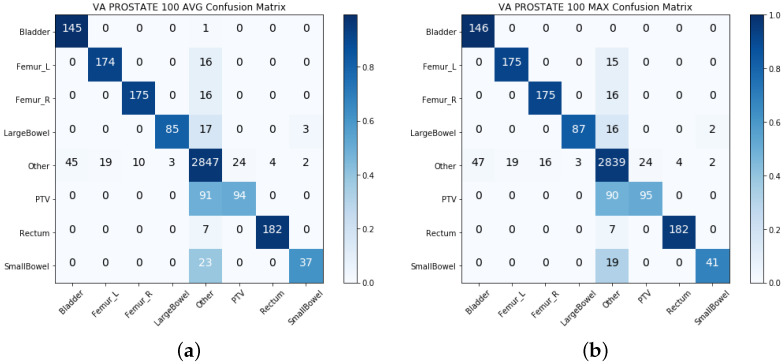

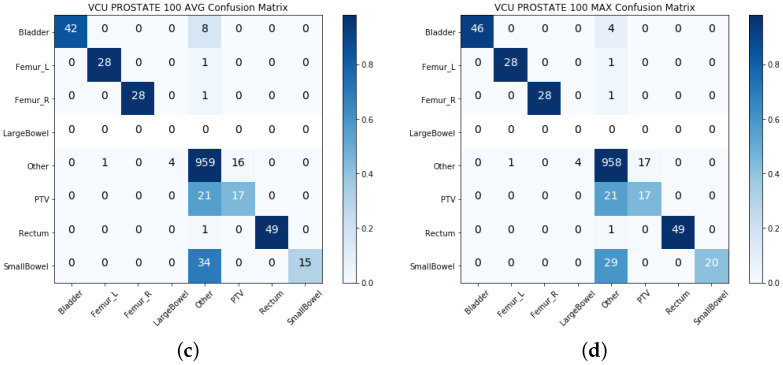
Late integration: confusion matrix for VA-ROQS and VCU prostate datasets: (**a**) VA-ROQS prostate AVG integration, (**b**) VA-ROQS prostate MAX integration, (**c**) VCU prostate AVG integration, and (**d**) VCU prostate MAX integration. A darker color demonstrates more accurate prediction, and the diagonal shows the labels predicted correctly.

Table 4 shows the macro-averaged precision, recall, and F${}_{1}$-scores. We noticed that the precision from the late integration by the MAX probability selection method was better than the single-view models for both the prostate and lung VCU datasets. However, the recall and F${}_{1}$-score dropped in this case. We also observed that the precision from the MAX for the VA-ROQS prostate dataset method was increased by 0.07, but the recall and F${}_{1}$-score were negatively affected. Overall, late integration with MAX had a negative effect on the VA-ROQS dataset. Figure 6 and Figure 7 show the confusion matrices for the lung and prostate dataset, respectively. Tables S9–S12 in the Supplementary Materials show the late integration results for the VA-ROQS and VCU data for both diseases. We observed that the random forest algorithm with the top 100 geometric features (as opposed to 50 features for the intermediate integration model) and the supervised fastText classifier with a 200 document vector size performed best. Tables S21–S24 in the Supplementary Materials show the label-wise late integration model results on all four datasets.

## 5. Discussion

In our previous work, we presented structure name standardization using the fastText supervised classification algorithm [3]. Although our model performed well, we observed some false positives. Our analysis showed that this was due to using the same labels for different structures across multiple treatment centers. For example, we observed that some radiation oncologists used Bowel to label SmallBowel, while others used it to label LargeBowel. Physician-specific preference might still be consistent at the center level. Such preferences create confusion when several patients’ data are pooled from various treatment centers to build the model. To address such issues, we investigated the use of the geometric information of structures for automatically identifying the standard structure names [8]. It was evident from the results that geometric information alone was not enough. Hence, we investigated the different approaches to integrating the physician-given names (textual labels) and the structures’ geometric information. We posited that geometric information would provide a different view of the structures, which would help differentiate structures when physician-given names are the same. Since we had different views (text and image) of the same structures, we integrated the different views at the machine learning pipeline’s intermediate and late stages.

### 5.1. Strengths and Limitations

This proposed novel approach standardizes radiotherapy structure names using the heterogeneous prostate and lung radiotherapy structures. We demonstrated that the multi-view integration approach improved the standardization process. Structure delineation generates significantly imbalanced datasets, but our approach can overcome data imbalance issues and hence can work well on real-world datasets.

The limitations of the proposed approach can be divided into clinical and methodological categories, as discussed below.

#### 5.1.1. Clinical Limitations

- So far, we were able to identify only OARs and PTV structures. Although these are critical structures, radiotherapy treatment involves other types of structures, such as GTV, CTV, and other derived structures. To fully standardize the data, we need to standardize all structures, not just the OARs and PTV.
- The OARs were originally selected based on the requirements of the VA-ROQS project, whose primary focus was treatment quality assessment based on specific quality metrics [9]. However, radiation oncologists may also delineate many other OAR structures, such as the kidney and liver. To truly build a generalized system to identify all possible structures, the dataset needs to identify all correctly labeled OAR structures, not just the significant OAR structures.

#### 5.1.2. Methodological Limitations

- Extraction of 3D volumes of structures requires selecting the bounding box size to make sure it covers the biggest possible structure in any given disease. Although it only needs to be done once per dataset, it adds to the overhead of the standardization pipeline.
- It is difficult to capture the image semantics by turning images into a single vector and taking the 50 or 100 top components from it.
- We extracted the structures fitted with bounding boxes. Using just structures and discarding the other surrounding structures and anatomical information may negatively affect the model performance.
- In late integration, we tested only AVG and MAX for combining the data, which gives equal importance to both the text and geometric data. As we saw that the single-view results on the geometric information model were performing poorly when compared to the text-based single-view model, a weighted average technique may produce better results.

### 5.2. Comparison With Previous Work

Our proposed approaches are fundamentally different from the current state-of-the-art in the literature. A 99% accuracy in structure name standardization was reported in [4]; however, their pipeline required manual labeling from clinicians coming from the same institution from which the datasets were generated. We achieved a similar accuracy even with multi-institute data. A couple other works used geometric information from DICOM-RT structure files and applied ML-based techniques for standardizing structure names [6,7]; although they achieved high accuracy, they used only the OAR structures and not the other ones. We however considered all the possible structures and demonstrated our performance on actual clinical data coming from multiple institutions (both federal and academic). Due to these differences, we could not compare the performance of our models with those from the literature. However, our multi-view approach is the first such attempt to integrate different views of data (textual and image) from a single patient for automatic standardization of arbitrary physician-given structure names.

### 5.3. Future Work

In this paper, we presented different methods to integrate the heterogeneous radiotherapy structure data for structure name standardization. We next outline the following future works for the structure name standardization problem.

- In this work, we focused on identifying only the standard structure names of some OARs and PTV structures. In the future, we would like to define the hierarchy of structures representing the logical groupings such as OARs, targets (PTV, CTV, and GTV), implants, derived OARs, and derived targets.
- Here, we used only the prostate and lung disease sites. We started with these sites because they cover the majority of the patients treated across the 40 different VA radiation therapy treatment centers. Moving forward, we would like to investigate the efficacy of our approach on other disease sites such as brain, head and neck, and abdomen, which make up smaller datasets compared to the prostate and lung disease sites.
- The current dataset was comprised of only CT images, whereas in practice, both CT and MRI image datasets are used for treatment planning. We will investigate the efficacy of our approach on both the CT and MRI datasets in the future.
- In the late integration approach, we used the top 100 SVD features with a random forest classification algorithm. However, there are more suitable algorithms for image data such as 2D CNN algorithm, ResNet [20], VoxNet, and a 3D CNN supervised classification algorithm [21]. The radiotherapy structure set is 3D in nature, making it more suitable to solve using 3D algorithms.
- The current list of OARs identified for both lung and prostate datasets is per the VA-ROQS project requirement, which selected these OARs in consensus with a team of experts. Radiation oncologists also delineate other types of OARs for each patient, such as kidney (left and right) and liver. Although these are not critical OARs in prostate cancer treatment, we believe building a system to identify and standardize all structures delineated according to the TG-263 guideline would provide the radiation therapy healthcare institutes with an opportunity to produce a robust dataset for downstream analysis projects. In this regard, an interesting future direction is to define hierarchy such as targets, OARs, implants, sub-target volumes (e.g. PTVminusCTV), sub-target OARs (e.g., rectum sub CTV), isodose structures, “ghost” structures for optimization, etc., and assess the efficiency of such ML models considering each of these different categories.

## 6. Conclusions

In this paper, we presented two types of multi-view data integration methods: intermediate and late integration for structure name standardization. We utilized the physician-given structure names and the geometric information of structures to build multi-view data integration methods. We observed that the intermediate integration methods improved the models’ overall performance, while late integration helped reduce the false negatives (higher precision). We validated our approach by training it on data from 30 VA RT centers and tested it on 10 VA radiotherapy centers and the VCU dataset. We showcased our model’s generalizability by demonstrating the higher accuracies on data coming from multiple institutions and also different types of cancers. We believe that the multi-view integration methods are very well suited for structure name standardization, as they make the best use of different information to avoid confusion. High model performance on the VA-ROQS test set showed that our approaches could generalize very well within the VA system. Furthermore, the excellent performance on the VCU dataset suggests the model’s ability to generalize well on the data from outside of the VA system.

## Figures and Tables

**Table 1:** Examples of physician-given structures’ names. OARs, Organs-At-Risk. Structure names in Patient_1, Patient_2, and Patient_3 show the variability in naming practices for structures.

Structure Type	Standard Name	Patient 1	Patient 1	Patient 1
OAR	Large Bowel	bowel_lg	colon	bowel
OAR	Femur_L	LtFemoral Head	Left Fem	Fem hdneck Lt
OAR	Femur_R	Fem Rt	Rt_Fem	Femoral_Rt
OAR	Bladder	bldr	bladder-KS	BLADDER
OAR	Small Bowel	bowel	SM_bowel	
OAR	Rectum	Rectum	Rect	rectum
Target	PTV	PTV_Prost	CTV	PTV
Other		Rectum subptv	Dose 107.1[%]	RFH
Other		Prostate	PTV79.2	Balloon
Other		PTV45	CTV45_OPT	CouchSurface
Other		CTV45	ProxSV	Pelvic Nodes
Other		Rec50	CTV vessels	Marker1
Other		POST	BLDSPARE	PROS+SV’S
Other		out	70opti	Blad_NO_ptv
Other		External	ROI_1	dosavoid2
Other		calcification	ROI_3	Marker3
Other		FIDUCIALS	bulb	External
Other		PTV_NDS	CouchInterior	Seeds

**Table 2:** Distribution of types of structures for prostate and lung cancer patients in the VA-ROQS and VCU datasets.

	VA-ROQS		VCU	
	Non-Standard Name		Non-Standard Name	
**Standard Name**	**Structure Count**	**Unique Count**	**Structure Count**	**Unique Count**
Brachial_Plexus	108	44	4	4
Esophagus	613	26	47	3
Heart	670	20	45	2
Other (Lung)	10,292	3,639	775	317
SpinalCord	681	37	48	6
PTV (Lung)	680	286	36	4
**Lung Total**	**13,044**	**4052**	**955**	**336**
Bladder	609	10	50	3
Femur_R	700	62	29	14
Femur_L	694	59	29	13
Rectum	719	14	50	3
SmallBowel	250	40	49	10
LargeBowel	341	34	0	0
Other (Prostate)	11,038	2799	980	434
PTV (Prostate)	714	236	38	16
**Prostate Total**	**15,065**	**3254**	**1225**	**493**
**Grand Totals**	**28,109**	**7306**	**2180**	**829**

**Table 3:** Intermediate integration—disease-specific macro-averaged precision, recall, F${}_{1}$-score, and overall accuracy. MLB: Majority Label Baseline.

Dataset	Disease	Data Type	Precision	Recall	F${}_{1}$-Score	Acc
Test (VA-ROQS)	Prostate	MLB	0.090	0.120	0.110	0.730
		Text	**0.890**	0.866	0.872	0.930
		Image	0.758	0.579	0.619	0.856
		Combined	0.874	**0.895**	**0.879**	**0.936**
	Lung	MLB	0.130	0.170	0.150	0.780
		Text	**0.921**	**0.874**	**0.893**	**0.950**
		Image	0.825	0.694	0.708	0.916
		Combined	0.896	0.873	0.882	0.946
Text (VCU)	Prostate	MLB	0.110	0.140	0.130	0.800
		Text	0.778	0.730	0.740	0.927
		Image	0.710	0.476	0.519	0.870
		Combined	**0.781**	**0.747**	**0.754**	**0.930**
	Lung	MLB	0.140	0.170	0.150	0.810
		Text	**0.830**	**0.981**	**0.873**	**0.969**
		Image	0.610	0.565	0.585	0.918
		Combined	0.821	0.976	0.860	0.964

**Table 4:** Late integration—disease-specific macro-averaged precision, recall, F${}_{1}$-score, and overall accuracy. MLB: Majority Label Baseline.

Dataset	Disease	Data Type	Precision	Recall	F${}_{1}$-Score	Acc
Test (VCU)	Prostate	MLB	0.110	0.140	0.130	0.800
		Text	0.778	**0.730**	**0.740**	0.927
		Image	0.710	0.476	0.519	0.870
		Avg	0.802	0.685	0.719	0.929
		Max	**0.801**	0.708	0.739	**0.936**
	Lung	MLB	0.140	0.170	0.150	0.810
		Text	0.830	**0.981**	**0.873**	**0.969**
		Image	0.610	0.565	0.585	0.918
		Avg	0.858	0.807	0.811	0.964
		Max	**0.849**	0.810	0.806	0.963
Test (VA-ROQS)	Prostate	MLB	0.090	0.120	0.110	0.730
		Text	0.890	**0.866**	**0.872**	0.930
		Image	0.758	0.579	0.619	0.856
		Avg	**0.897**	0.836	0.857	0.930
		Max	**0.897**	0.848	0.864	**0.930**
	Lung	MLB	0.130	0.170	0.150	0.780
		Text	**0.921**	**0.874**	**0.893**	**0.950**
		Image	0.825	0.694	0.708	0.916
		Avg	0.918	0.840	0.868	0.964
		Max	0.916	0.840	0.867	0.945

## Data Availability

No new data were created or analyzed in this study. Data sharing is not applicable to this article.

## Supplementary Materials

The following are available online at https://www.mdpi.com/article/10.3390/cancers13081796/s1, Table S1: Single-view with image features model results for VA-ROQS, lung disease-specific macro-averaged precision, recall, F${}_{1}$-score, and overall accuracy; Table S2: Single-view with image features model results for VA-ROQS, prostate disease specific macro-averaged precision, recall, F${}_{1}$-score, and overall accuracy; Table S3: Single-view with image features model results for VCU data lung disease specific macro-averaged precision, recall, F${}_{1}$-score, and overall accuracy; Table S4: Single-view with image features model results for VCU data prostate disease specific macro-averaged precision, recall, F${}_{1}$-score, and overall accuracy; Table S5: Intermediate integration results—VA-ROQS data lung disease-specific macro-averaged precision, recall, F${}_{1}$-score, and overall accuracy; Table S6: Intermediate integration results—VA-ROQS dataset prostate disease-specific macro-averaged precision, recall, F${}_{1}$-score, and overall accuracy; Table S7: Intermediate integration results—VCU dataset lung disease-specific macro-averaged precision, recall, F${}_{1}$-score, and overall accuracy; Table S8: Intermediate integration results—VCU dataset prostate disease-specific macro-averaged precision, recall, F${}_{1}$-score, and overall accuracy; Table S9: Late integration results—VA-ROQS dataset lung disease-specific macro-averaged precision, recall, F${}_{1}$-score, and overall accuracy; Table S10: Late integration results—VA-ROQS dataset prostate disease-specific macro-averaged precision, recall, F${}_{1}$-score, and overall accuracy; Table S11: Late integration results—VCU dataset prostate disease-specific macro-averaged precision, recall, F${}_{1}$-score, and overall accuracy; Table S12: Late integration results—VCU dataset lung disease-specific macro-averaged precision, recall, F${}_{1}$-score, and overall accuracy; Table S13: Single-view label-wise results—VA-ROQS dataset Lung, BonesSeparate, with 100 image features; Table S14: Single-view label-wise results—VA-ROQS dataset prostate, BonesSeparate, with 100 image features; Table S15: Single-view label-wise results—VCU dataset lung, BonesSeparate, with 100 image features; Table S16: Single-view label-wise results—VCU dataset prostate, BonesSeparate, with 100 image features; Table S17: Intermediate integration label-wise results—VA-ROQS dataset lung, BonesSeparate, 50 image features, 200 text features, with random forest algorithm; Table S18: Intermediate integration label-wise results—VCU dataset Lung, BonesSeparate, 50 image features, 200 text features, with random forest algorithm; Table S19: Intermediate integration label-wise results—VA-ROQS dataset prostate, BonesSeparate, 50 image features, 200 text features, with random forest algorithm; Table S20: Intermediate integration label-wise results—VCU dataset prostate, BonesSeparate, 50 image features, 200 text features, with random forest algorithm; Table S21: Late integration label-wise results—VA-ROQS dataset lung, 100 image features, 200 text features; Table S22: Late integration label-wise results—VCU dataset lung, BonesSeparate, 100 image features, 200 text features; Table S23: Late integration label-wise results—VA-ROQS dataset prostate, BonesSeparate, 100 image features, 200 text features; Table S24:Late integration label-wise results—VCU dataset prostate, BonesSeparate, 100 image features, 200 text features.

## Abbreviations

The following abbreviations are used in this manuscript:

AAPM	American Association of Physicists in Medicine
ASTRO	American Society for Radiation Oncology
TG-263	Task Group-263
RT	Radiotherapy
ROQS	Radiation Oncology Quality Surveillance Program
VCU	Virginia Commonwealth University
VA	Veterans Health Administration
OARs	Organs-At-Risk
Non_OARs	Non Organs-At-Risk
CT	Computed Tomography
MRI	Magnetic Resonance Imaging
HU	Hounsfield Unit
PRV	Planning Organs-At-Risk Volume
PTV	Planning Target Volume
GTV	Gross Target Volume
CTV	Clinical Target Volume
DVH	Dose Volume Histogram
DICOM	Digital Imaging and Communication in Medicine
QA	Quality Assurance
NLP	Natural Language Processing
ML	Machine Learning
SVD	Singular-Value Decomposition
TPS	Treatment Planning System
SVM	Support Vector Machine
KNN	k-Nearest Neighbors
RF	Random Forest
PCA	Principal Component Analysis
