# Supplementary material for: Multi-View Data Integration Methods for Radiotherapy Structure Name Standardization
Source: Cancers (Basel). 2021 Apr 9;13(8):1796. doi: 10.3390/cancers13081796 (PMC8070367; doi:10.3390/cancers13081796)
Supplement: Supplementary file 1 [file cancers-13-01796-s001.pdf]

Supplementary Materials: Multi-View Data Integration Methods for Radiotherapy Structure Name Standardization

Khajamoinuddin Syed [1], William C. Sleeman IV [1,2], Michael Hagan [2,3], Jatinder Palta [2,3], Rishabh Kapoor[2,3], and Preetam Ghosh[1]

Table 1. Single-View with Image Features Model Results for VA-ROQS, Lung Disease specific macro-averaged Precision, Recall, F_1-Score, and Overall Accuracy.

Dataset	Disease	Image Features Type	Image Feature Size	Algorithm	Precision	Recall	F_1-Score	Acc	Support
VA	lung	BonesSeparate	50	RF	0.765	0.673	0.681	0.914	2564
VA	lung	BonesSeparate	50	LR	0.751	0.628	0.646	0.888	2564
VA	lung	BonesSeparate	50	SVM_Linear	0.63	0.598	0.595	0.89	2564
VA	lung	BonesSeparate	50	SVM_RBF	0.598	0.635	0.616	0.911	2564
VA	lung	BonesSeparate	100	RF	0.825	0.694	0.708	0.916	2564
VA	lung	BonesSeparate	100	LR	0.729	0.69	0.693	0.887	2564
VA	lung	BonesSeparate	100	SVM_Linear	0.654	0.658	0.651	0.888	2564
VA	lung	BonesSeparate	100	SVM_RBF	0.725	0.717	0.716	0.911	2564
VA	lung	BonesSeparate	250	RF	0.91	0.667	0.693	0.91	2564
VA	lung	BonesSeparate	250	LR	0.73	0.723	0.719	0.887	2564
VA	lung	BonesSeparate	250	SVM_Linear	0.66	0.704	0.68	0.896	2564
VA	lung	BonesSeparate	250	SVM_RBF	0.716	0.713	0.711	0.91	2564
VA	lung	BonesSeparate	500	RF	0.708	0.625	0.651	0.904	2564
VA	lung	BonesSeparate	500	LR	0.657	0.732	0.682	0.864	2564
VA	lung	BonesSeparate	500	SVM_Linear	0.634	0.743	0.663	0.879	2564
VA	lung	BonesSeparate	500	SVM_RBF	0.719	0.705	0.709	0.908	2564
VA	lung	BonesSeparate	1000	RF	0.783	0.572	0.602	0.897	2564
VA	lung	BonesSeparate	1000	LR	0.605	0.734	0.657	0.831	2564
VA	lung	BonesSeparate	1000	SVM_Linear	0.603	0.722	0.645	0.826	2564
VA	lung	BonesSeparate	1000	SVM_RBF	0.725	0.693	0.705	0.907	2564
VA	lung	NoBones	50	LR	0.544	0.487	0.491	0.844	2564
VA	lung	NoBones	50	SVM_Linear	0.372	0.415	0.392	0.835	2564
VA	lung	NoBones	50	SVM_RBF	0.547	0.578	0.562	0.892	2564
VA	lung	NoBones	100	RF	0.703	0.644	0.658	0.904	2564
VA	lung	NoBones	100	LR	0.566	0.548	0.544	0.854	2564
VA	lung	NoBones	100	SVM_Linear	0.518	0.534	0.525	0.862	2564
VA	lung	NoBones	100	SVM_RBF	0.56	0.578	0.567	0.894	2564
VA	lung	NoBones	250	RF	0.729	0.646	0.666	0.905	2564
VA	lung	NoBones	250	LR	0.608	0.618	0.605	0.86	2564
VA	lung	NoBones	250	SVM_Linear	0.54	0.584	0.561	0.875	2564
VA	lung	NoBones	250	SVM_RBF	0.573	0.59	0.58	0.895	2564
VA	lung	NoBones	500	RF	0.749	0.605	0.626	0.9	2564
VA	lung	NoBones	500	LR	0.591	0.628	0.604	0.844	2564
VA	lung	NoBones	500	SVM_Linear	0.592	0.594	0.572	0.862	2564
VA	lung	NoBones	500	SVM_RBF	0.578	0.58	0.578	0.894	2564
VA	lung	NoBones	1000	RF	0.754	0.573	0.591	0.896	2564
VA	lung	NoBones	1000	LR	0.536	0.618	0.564	0.797	2564
VA	lung	NoBones	1000	SVM_Linear	0.543	0.634	0.563	0.781	2564
VA	lung	NoBones	1000	SVM_RBF	0.594	0.564	0.577	0.894	2564

Table 2. Single-View with Image Features Model Results for VA-ROQS, Prostate Disease specific macro-averaged Precision, Recall, F_1-Score, and Overall Accuracy.

Dataset	Disease	Image Features Type	Image Feature Size	Algorithm	Precision	Recall	F_1-Score	Acc	Support
VA	prostate	BonesSeparate	50	RF	0.747	0.608	0.645	0.858	4020
VA	prostate	BonesSeparate	50	LR	0.701	0.632	0.65	0.852	4020
VA	prostate	BonesSeparate	50	SVM_Linear	0.64	0.653	0.642	0.855	4020
VA	prostate	BonesSeparate	50	SVM_RBF	0.681	0.722	0.697	0.874	4020
VA	prostate	BonesSeparate	100	RF	0.758	0.579	0.619	0.856	4020
VA	prostate	BonesSeparate	100	LR	0.721	0.674	0.686	0.861	4020
VA	prostate	BonesSeparate	100	SVM_Linear	0.671	0.676	0.67	0.866	4020
VA	prostate	BonesSeparate	100	SVM_RBF	0.705	0.709	0.703	0.879	4020
VA	prostate	BonesSeparate	250	RF	0.68	0.509	0.547	0.85	4020
VA	prostate	BonesSeparate	250	LR	0.654	0.671	0.657	0.832	4020
VA	prostate	BonesSeparate	250	SVM_Linear	0.632	0.686	0.654	0.85	4020
VA	prostate	BonesSeparate	250	SVM_RBF	0.708	0.669	0.684	0.874	4020
VA	prostate	BonesSeparate	500	RF	0.718	0.46	0.502	0.841	4020
VA	prostate	BonesSeparate	500	LR	0.592	0.657	0.618	0.795	4020
VA	prostate	BonesSeparate	500	SVM_Linear	0.615	0.681	0.64	0.803	4020
VA	prostate	BonesSeparate	500	SVM_RBF	0.725	0.605	0.646	0.868	4020
VA	prostate	BonesSeparate	1000	RF	0.642	0.402	0.44	0.828	4020
VA	prostate	BonesSeparate	1000	LR	0.572	0.626	0.591	0.775	4020
VA	prostate	BonesSeparate	1000	SVM_Linear	0.601	0.667	0.623	0.781	4020
VA	prostate	BonesSeparate	1000	SVM_RBF	0.758	0.507	0.565	0.855	4020
VA	prostate	NoBones	50	RF	0.73	0.55	0.6	0.845	4020
VA	prostate	NoBones	50	LR	0.694	0.545	0.59	0.834	4020
VA	prostate	NoBones	50	SVM_Linear	0.651	0.515	0.542	0.834	4020
VA	prostate	NoBones	50	SVM_RBF	0.672	0.566	0.591	0.854	4020
VA	prostate	NoBones	100	RF	0.739	0.539	0.59	0.847	4020
VA	prostate	NoBones	100	LR	0.688	0.606	0.631	0.846	4020
VA	prostate	NoBones	100	SVM_Linear	0.644	0.574	0.597	0.841	4020
VA	prostate	NoBones	100	SVM_RBF	0.718	0.566	0.6	0.858	4020
VA	prostate	NoBones	250	RF	0.705	0.499	0.555	0.841	4020
VA	prostate	NoBones	250	LR	0.65	0.628	0.63	0.832	4020
VA	prostate	NoBones	250	SVM_Linear	0.595	0.602	0.595	0.843	4020
VA	prostate	NoBones	250	SVM_RBF	0.729	0.55	0.591	0.855	4020
VA	prostate	NoBones	500	RF	0.723	0.448	0.507	0.833	4020
VA	prostate	NoBones	500	LR	0.618	0.619	0.612	0.812	4020
VA	prostate	NoBones	500	SVM_Linear	0.602	0.6	0.594	0.808	4020
VA	prostate	NoBones	500	SVM_RBF	0.646	0.504	0.549	0.845	4020
VA	prostate	NoBones	1000	RF	0.695	0.409	0.468	0.823	4020
VA	prostate	NoBones	1000	LR	0.574	0.59	0.578	0.786	4020
VA	prostate	NoBones	1000	SVM_Linear	0.542	0.579	0.557	0.776	4020
VA	prostate	NoBones	1000	SVM_RBF	0.651	0.453	0.511	0.834	4020

Table 3. Single-View with Image Features Model Results for VCU data Lung Disease specific macro-averaged Precision, Recall, F_1-Score, and Overall Accuracy.

Dataset	Disease	Image Features Type	Image Feature Size	Algorithm	Precision	Recall	F_1-Score	Acc	Support
VCU	lung	BonesSeparate	50	RF	0.635	0.598	0.614	0.932	955
VCU	lung	BonesSeparate	50	LR	0.543	0.55	0.538	0.866	955
VCU	lung	BonesSeparate	50	SVM_Linear	0.519	0.524	0.519	0.874	955
VCU	lung	BonesSeparate	50	SVM_RBF	0.565	0.61	0.584	0.908	955
VCU	lung	BonesSeparate	100	RF	0.61	0.565	0.585	0.918	955
VCU	lung	BonesSeparate	100	LR	0.587	0.579	0.568	0.871	955
VCU	lung	BonesSeparate	100	SVM_Linear	0.526	0.587	0.548	0.865	955
VCU	lung	BonesSeparate	100	SVM_RBF	0.604	0.647	0.621	0.904	955
VCU	lung	BonesSeparate	250	RF	0.629	0.552	0.583	0.919	955
VCU	lung	BonesSeparate	250	LR	0.573	0.643	0.584	0.857	955
VCU	lung	BonesSeparate	250	SVM_Linear	0.552	0.619	0.573	0.88	955
VCU	lung	BonesSeparate	250	SVM_RBF	0.583	0.626	0.599	0.895	955
VCU	lung	BonesSeparate	500	RF	0.633	0.541	0.579	0.917	955
VCU	lung	BonesSeparate	500	LR	0.518	0.63	0.546	0.807	955
VCU	lung	BonesSeparate	500	SVM_Linear	0.521	0.636	0.542	0.838	955
VCU	lung	BonesSeparate	500	SVM_RBF	0.595	0.613	0.598	0.895	955
VCU	lung	BonesSeparate	1000	RF	0.621	0.494	0.542	0.903	955
VCU	lung	BonesSeparate	1000	LR	0.499	0.624	0.542	0.791	955
VCU	lung	BonesSeparate	1000	SVM_Linear	0.529	0.662	0.552	0.792	955
VCU	lung	BonesSeparate	1000	SVM_RBF	0.566	0.565	0.559	0.896	955
VCU	lung	NoBones	50	RF	0.713	0.569	0.603	0.913	955
VCU	lung	NoBones	50	LR	0.474	0.448	0.448	0.836	955
VCU	lung	NoBones	50	SVM_Linear	0.342	0.381	0.358	0.816	955
VCU	lung	NoBones	50	SVM_RBF	0.539	0.547	0.542	0.907	955
VCU	lung	NoBones	100	RF	0.563	0.528	0.543	0.914	955
VCU	lung	NoBones	100	LR	0.5	0.5	0.491	0.845	955
VCU	lung	NoBones	100	SVM_Linear	0.471	0.51	0.487	0.855	955
VCU	lung	NoBones	100	SVM_RBF	0.534	0.539	0.533	0.897	955
VCU	lung	NoBones	250	RF	0.582	0.531	0.553	0.915	955
VCU	lung	NoBones	250	LR	0.489	0.511	0.497	0.832	955
VCU	lung	NoBones	250	SVM_Linear	0.485	0.518	0.499	0.844	955
VCU	lung	NoBones	250	SVM_RBF	0.525	0.533	0.527	0.888	955
VCU	lung	NoBones	500	RF	0.595	0.537	0.562	0.917	955
VCU	lung	NoBones	500	LR	0.483	0.496	0.488	0.815	955
VCU	lung	NoBones	500	SVM_Linear	0.477	0.513	0.491	0.825	955
VCU	lung	NoBones	500	SVM_RBF	0.524	0.515	0.517	0.885	955
VCU	lung	NoBones	1000	RF	0.609	0.523	0.557	0.914	955
VCU	lung	NoBones	1000	LR	0.453	0.519	0.478	0.773	955
VCU	lung	NoBones	1000	SVM_Linear	0.492	0.529	0.498	0.758	955
VCU	lung	NoBones	1000	SVM_RBF	0.53	0.509	0.517	0.887	955

Table 4. Single-View with Image Features Model Results for VCU data Prostate Disease specific macro-averaged Precision, Recall, F_1-Score, and Overall Accuracy.

Dataset	Disease	Image Features Type	Image Feature Size	Algorithm	Precision	Recall	F_1-Score	Acc	Support
VCU	prostate	BonesSeparate	50	RF	0.73	0.508	0.552	0.878	1225
VCU	prostate	BonesSeparate	50	LR	0.675	0.528	0.557	0.869	1225
VCU	prostate	BonesSeparate	50	SVM_Linear	0.662	0.51	0.528	0.868	1225
VCU	prostate	BonesSeparate	50	SVM_RBF	0.691	0.516	0.548	0.878	1225
VCU	prostate	BonesSeparate	100	RF	0.71	0.476	0.519	0.87	1225
VCU	prostate	BonesSeparate	100	LR	0.672	0.538	0.565	0.866	1225
VCU	prostate	BonesSeparate	100	SVM_Linear	0.663	0.525	0.542	0.871	1225
VCU	prostate	BonesSeparate	100	SVM_RBF	0.692	0.519	0.545	0.877	1225
VCU	prostate	BonesSeparate	250	RF	0.711	0.455	0.498	0.869	1225
VCU	prostate	BonesSeparate	250	LR	0.651	0.564	0.591	0.86	1225
VCU	prostate	BonesSeparate	250	SVM_Linear	0.589	0.532	0.537	0.851	1225
VCU	prostate	BonesSeparate	250	SVM_RBF	0.693	0.506	0.532	0.874	1225
VCU	prostate	BonesSeparate	500	RF	0.693	0.435	0.463	0.864	1225
VCU	prostate	BonesSeparate	500	LR	0.6	0.534	0.554	0.839	1225
VCU	prostate	BonesSeparate	500	SVM_Linear	0.635	0.581	0.593	0.842	1225
VCU	prostate	BonesSeparate	500	SVM_RBF	0.689	0.494	0.517	0.872	1225
VCU	prostate	BonesSeparate	1000	RF	0.667	0.429	0.456	0.849	1225
VCU	prostate	BonesSeparate	1000	LR	0.526	0.482	0.497	0.797	1225
VCU	prostate	BonesSeparate	1000	SVM_Linear	0.629	0.52	0.555	0.807	1225
VCU	prostate	BonesSeparate	1000	SVM_RBF	0.704	0.466	0.494	0.869	1225
VCU	prostate	NoBones	50	RF	0.69	0.441	0.493	0.857	1225
VCU	prostate	NoBones	50	LR	0.639	0.442	0.49	0.851	1225
VCU	prostate	NoBones	50	SVM_Linear	0.633	0.432	0.482	0.863	1225
VCU	prostate	NoBones	50	SVM_RBF	0.675	0.435	0.484	0.867	1225
VCU	prostate	NoBones	100	RF	0.679	0.431	0.479	0.86	1225
VCU	prostate	NoBones	100	LR	0.635	0.493	0.543	0.844	1225
VCU	prostate	NoBones	100	SVM_Linear	0.596	0.461	0.506	0.86	1225
VCU	prostate	NoBones	100	SVM_RBF	0.675	0.437	0.487	0.866	1225
VCU	prostate	NoBones	250	RF	0.68	0.398	0.447	0.853	1225
VCU	prostate	NoBones	250	LR	0.561	0.489	0.509	0.827	1225
VCU	prostate	NoBones	250	SVM_Linear	0.557	0.48	0.5	0.841	1225
VCU	prostate	NoBones	250	SVM_RBF	0.674	0.429	0.472	0.863	1225
VCU	prostate	NoBones	500	RF	0.71	0.399	0.45	0.854	1225
VCU	prostate	NoBones	500	LR	0.5	0.464	0.475	0.795	1225
VCU	prostate	NoBones	500	SVM_Linear	0.514	0.482	0.489	0.789	1225
VCU	prostate	NoBones	500	SVM_RBF	0.669	0.406	0.452	0.857	1225
VCU	prostate	NoBones	1000	RF	0.583	0.362	0.414	0.85	1225
VCU	prostate	NoBones	1000	LR	0.442	0.464	0.44	0.76	1225
VCU	prostate	NoBones	1000	SVM_Linear	0.472	0.471	0.451	0.757	1225
VCU	prostate	NoBones	1000	SVM_RBF	0.543	0.364	0.412	0.848	1225

Table 5. Intermediate Integration Results - VA-ROQS data Lung Disease specific macro-averaged Precision, Recall, F_1-Score, and Overall Accuracy.

Dataset	Disease	Image Features Type	Image Feature Size	Algorithm	Precision	Recall	F_1-Score	Acc	Support
VA	lung	BonesSeparate	50	RF	0.896	0.873	0.882	0.946	2564
VA	lung	BonesSeparate	50	LR	0.896	0.807	0.84	0.945	2564
VA	lung	BonesSeparate	50	SVM_Linear	0.939	0.741	0.778	0.943	2564
VA	lung	BonesSeparate	50	SVM_RBF	0.889	0.87	0.871	0.946	2564
VA	lung	BonesSeparate	100	RF	0.885	0.867	0.873	0.943	2564
VA	lung	BonesSeparate	100	LR	0.9	0.828	0.856	0.946	2564
VA	lung	BonesSeparate	100	SVM_Linear	0.893	0.758	0.797	0.944	2564
VA	lung	BonesSeparate	100	SVM_RBF	0.885	0.804	0.834	0.943	2564
VA	lung	BonesSeparate	250	RF	0.892	0.869	0.878	0.946	2564
VA	lung	BonesSeparate	250	LR	0.899	0.826	0.854	0.945	2564
VA	lung	BonesSeparate	250	SVM_Linear	0.902	0.801	0.836	0.945	2564
VA	lung	BonesSeparate	250	SVM_RBF	0.885	0.766	0.803	0.943	2564
VA	lung	BonesSeparate	500	RF	0.887	0.869	0.876	0.944	2564
VA	lung	BonesSeparate	500	LR	0.883	0.825	0.849	0.943	2564
VA	lung	BonesSeparate	500	SVM_Linear	0.897	0.834	0.857	0.946	2564
VA	lung	BonesSeparate	500	SVM_RBF	0.881	0.764	0.801	0.942	2564
VA	lung	BonesSeparate	1000	RF	0.886	0.868	0.874	0.944	2564
VA	lung	BonesSeparate	1000	LR	0.861	0.844	0.85	0.938	2564
VA	lung	BonesSeparate	1000	SVM_Linear	0.881	0.833	0.851	0.943	2564
VA	lung	BonesSeparate	1000	SVM_RBF	0.878	0.756	0.792	0.943	2564
VA	lung	NoBones	50	RF	0.884	0.872	0.875	0.946	2564
VA	lung	NoBones	50	LR	0.916	0.813	0.85	0.944	2564
VA	lung	NoBones	50	SVM_Linear	0.774	0.715	0.733	0.943	2564
VA	lung	NoBones	50	SVM_RBF	0.889	0.859	0.868	0.945	2564
VA	lung	NoBones	100	RF	0.884	0.876	0.878	0.946	2564
VA	lung	NoBones	100	LR	0.917	0.815	0.852	0.945	2564
VA	lung	NoBones	100	SVM_Linear	0.774	0.715	0.733	0.943	2564
VA	lung	NoBones	100	SVM_RBF	0.911	0.777	0.818	0.943	2564
VA	lung	NoBones	250	RF	0.888	0.883	0.882	0.947	2564
VA	lung	NoBones	250	LR	0.901	0.815	0.847	0.943	2564
VA	lung	NoBones	250	SVM_Linear	0.774	0.715	0.733	0.943	2564
VA	lung	NoBones	250	SVM_RBF	0.773	0.714	0.732	0.942	2564
VA	lung	NoBones	500	RF	0.885	0.868	0.874	0.943	2564
VA	lung	NoBones	500	LR	0.884	0.821	0.846	0.942	2564
VA	lung	NoBones	500	SVM_Linear	0.937	0.723	0.749	0.942	2564
VA	lung	NoBones	500	SVM_RBF	0.774	0.712	0.73	0.942	2564
VA	lung	NoBones	1000	RF	0.881	0.871	0.874	0.944	2564
VA	lung	NoBones	1000	LR	0.885	0.824	0.849	0.943	2564
VA	lung	NoBones	1000	SVM_Linear	0.937	0.725	0.75	0.943	2564
VA	lung	NoBones	1000	SVM_RBF	0.773	0.71	0.728	0.941	2564

Table 6. Intermediate Integration Results - VA-ROQS dataset Prostate Disease specific macro-averaged Precision, Recall, F_1-Score, and Overall Accuracy.

Dataset	Disease	Image Features Type	Image Feature Size	Algorithm	Precision	Recall	F_1-Score	Acc	Support
VA	prostate	BonesSeparate	50	RF	0.874	0.895	0.879	0.936	4020
VA	prostate	BonesSeparate	50	LR	0.85	0.873	0.854	0.931	4020
VA	prostate	BonesSeparate	50	SVM_Linear	0.848	0.897	0.864	0.932	4020
VA	prostate	BonesSeparate	50	SVM_RBF	0.863	0.908	0.878	0.938	4020
VA	prostate	BonesSeparate	100	RF	0.875	0.893	0.879	0.935	4020
VA	prostate	BonesSeparate	100	LR	0.855	0.873	0.856	0.932	4020
VA	prostate	BonesSeparate	100	SVM_Linear	0.849	0.903	0.867	0.932	4020
VA	prostate	BonesSeparate	100	SVM_RBF	0.86	0.907	0.875	0.937	4020
VA	prostate	BonesSeparate	250	RF	0.867	0.896	0.875	0.932	4020
VA	prostate	BonesSeparate	250	LR	0.861	0.877	0.86	0.933	4020
VA	prostate	BonesSeparate	250	SVM_Linear	0.852	0.903	0.868	0.932	4020
VA	prostate	BonesSeparate	250	SVM_RBF	0.853	0.897	0.867	0.933	4020
VA	prostate	BonesSeparate	500	RF	0.86	0.903	0.875	0.932	4020
VA	prostate	BonesSeparate	500	LR	0.864	0.875	0.861	0.932	4020
VA	prostate	BonesSeparate	500	SVM_Linear	0.846	0.899	0.864	0.931	4020
VA	prostate	BonesSeparate	500	SVM_RBF	0.853	0.902	0.867	0.933	4020
VA	prostate	BonesSeparate	1000	RF	0.866	0.903	0.877	0.934	4020
VA	prostate	BonesSeparate	1000	LR	0.852	0.847	0.842	0.926	4020
VA	prostate	BonesSeparate	1000	SVM_Linear	0.847	0.888	0.859	0.929	4020
VA	prostate	BonesSeparate	1000	SVM_RBF	0.847	0.862	0.845	0.928	4020
VA	prostate	NoBones	50	RF	0.871	0.893	0.878	0.933	4020
VA	prostate	NoBones	50	LR	0.842	0.877	0.852	0.929	4020
VA	prostate	NoBones	50	SVM_Linear	0.846	0.898	0.863	0.93	4020
VA	prostate	NoBones	50	SVM_RBF	0.859	0.91	0.876	0.937	4020
VA	prostate	NoBones	100	RF	0.87	0.891	0.876	0.93	4020
VA	prostate	NoBones	100	LR	0.851	0.883	0.859	0.931	4020
VA	prostate	NoBones	100	SVM_Linear	0.848	0.896	0.864	0.93	4020
VA	prostate	NoBones	100	SVM_RBF	0.857	0.903	0.873	0.936	4020
VA	prostate	NoBones	250	RF	0.868	0.895	0.875	0.932	4020
VA	prostate	NoBones	250	LR	0.846	0.874	0.853	0.927	4020
VA	prostate	NoBones	250	SVM_Linear	0.848	0.902	0.865	0.93	4020
VA	prostate	NoBones	250	SVM_RBF	0.847	0.899	0.863	0.93	4020
VA	prostate	NoBones	500	RF	0.873	0.892	0.877	0.93	4020
VA	prostate	NoBones	500	LR	0.845	0.857	0.844	0.926	4020
VA	prostate	NoBones	500	SVM_Linear	0.841	0.891	0.858	0.928	4020
VA	prostate	NoBones	500	SVM_RBF	0.843	0.886	0.856	0.928	4020
VA	prostate	NoBones	1000	RF	0.868	0.896	0.876	0.934	4020
VA	prostate	NoBones	1000	LR	0.839	0.836	0.83	0.922	4020
VA	prostate	NoBones	1000	SVM_Linear	0.842	0.881	0.854	0.924	4020
VA	prostate	NoBones	1000	SVM_RBF	0.836	0.844	0.833	0.921	4020

Table 7. Intermediate Integration Results - VCU dataset Lung Disease specific macro-averaged Precision, Recall, F_1-Score, and Overall Accuracy.

Dataset	Disease	Image Features Type	Image Feature Size	Algorithm	Precision	Recall	F_1-Score	Acc	Support
VCU	lung	BonesSeparate	50	RF	0.821	0.976	0.86	0.964	955
VCU	lung	BonesSeparate	50	LR	0.832	0.977	0.874	0.968	955
VCU	lung	BonesSeparate	50	SVM_Linear	0.855	0.895	0.872	0.971	955
VCU	lung	BonesSeparate	50	SVM_RBF	0.817	0.981	0.861	0.964	955
VCU	lung	BonesSeparate	100	RF	0.82	0.985	0.865	0.966	955
VCU	lung	BonesSeparate	100	LR	0.811	0.939	0.853	0.964	955
VCU	lung	BonesSeparate	100	SVM_Linear	0.838	0.894	0.863	0.97	955
VCU	lung	BonesSeparate	100	SVM_RBF	0.818	0.935	0.858	0.965	955
VCU	lung	BonesSeparate	250	RF	0.823	0.986	0.869	0.968	955
VCU	lung	BonesSeparate	250	LR	0.802	0.943	0.85	0.962	955
VCU	lung	BonesSeparate	250	SVM_Linear	0.823	0.894	0.853	0.968	955
VCU	lung	BonesSeparate	250	SVM_RBF	0.838	0.894	0.863	0.97	955
VCU	lung	BonesSeparate	500	RF	0.817	0.981	0.861	0.964	955
VCU	lung	BonesSeparate	500	LR	0.811	0.939	0.858	0.963	955
VCU	lung	BonesSeparate	500	SVM_Linear	0.801	0.893	0.834	0.963	955
VCU	lung	BonesSeparate	500	SVM_RBF	0.817	0.852	0.831	0.966	955
VCU	lung	BonesSeparate	1000	RF	0.825	0.986	0.872	0.968	955
VCU	lung	BonesSeparate	1000	LR	0.799	0.92	0.846	0.956	955
VCU	lung	BonesSeparate	1000	SVM_Linear	0.799	0.934	0.847	0.959	955
VCU	lung	BonesSeparate	1000	SVM_RBF	0.795	0.851	0.818	0.958	955
VCU	lung	NoBones	50	RF	0.819	0.976	0.86	0.964	955
VCU	lung	NoBones	50	LR	0.824	0.981	0.869	0.966	955
VCU	lung	NoBones	50	SVM_Linear	0.827	0.853	0.836	0.97	955
VCU	lung	NoBones	50	SVM_RBF	0.817	0.981	0.861	0.964	955
VCU	lung	NoBones	100	RF	0.819	0.985	0.867	0.966	955
VCU	lung	NoBones	100	LR	0.82	0.981	0.867	0.965	955
VCU	lung	NoBones	100	SVM_Linear	0.827	0.853	0.836	0.97	955
VCU	lung	NoBones	100	SVM_RBF	0.839	0.977	0.887	0.968	955
VCU	lung	NoBones	250	RF	0.814	0.985	0.861	0.964	955
VCU	lung	NoBones	250	LR	0.804	0.984	0.857	0.961	955
VCU	lung	NoBones	250	SVM_Linear	0.823	0.853	0.834	0.969	955
VCU	lung	NoBones	250	SVM_RBF	0.827	0.853	0.836	0.97	955
VCU	lung	NoBones	500	RF	0.811	0.985	0.859	0.963	955
VCU	lung	NoBones	500	LR	0.801	0.984	0.853	0.96	955
VCU	lung	NoBones	500	SVM_Linear	0.827	0.853	0.836	0.97	955
VCU	lung	NoBones	500	SVM_RBF	0.771	0.812	0.789	0.971	955
VCU	lung	NoBones	1000	RF	0.82	0.985	0.865	0.966	955
VCU	lung	NoBones	1000	LR	0.806	0.98	0.854	0.961	955
VCU	lung	NoBones	1000	SVM_Linear	0.819	0.852	0.832	0.968	955
VCU	lung	NoBones	1000	SVM_RBF	0.764	0.811	0.785	0.969	955

Table 8. Intermediate Integration Results- VCU dataset Prostate Disease specific macro-averaged Precision, Recall, F_1-Score, and Overall Accuracy.

Dataset	Disease	Image Features Type	Image Feature Size	Algorithm	Precision	Recall	F_1-Score	Acc	Support
VCU	prostate	BonesSeparate	50	RF	0.781	0.747	0.754	0.93	1225
VCU	prostate	BonesSeparate	50	LR	0.780	0.772	0.771	0.939	1225
VCU	prostate	BonesSeparate	50	SVM_Linear	0.778	0.792	0.782	0.941	1225
VCU	prostate	BonesSeparate	50	SVM_RBF	0.779	0.771	0.769	0.934	1225
VCU	prostate	BonesSeparate	100	RF	0.789	0.777	0.775	0.938	1225
VCU	prostate	BonesSeparate	100	LR	0.776	0.762	0.761	0.934	1225
VCU	prostate	BonesSeparate	100	SVM_Linear	0.772	0.779	0.77	0.935	1225
VCU	prostate	BonesSeparate	100	SVM_RBF	0.78	0.774	0.771	0.936	1225
VCU	prostate	BonesSeparate	250	RF	0.78	0.770	0.767	0.934	1225
VCU	prostate	BonesSeparate	250	LR	0.774	0.749	0.751	0.927	1225
VCU	prostate	BonesSeparate	250	SVM_Linear	0.773	0.767	0.762	0.931	1225
VCU	prostate	BonesSeparate	250	SVM_RBF	0.773	0.769	0.764	0.931	1225
VCU	prostate	BonesSeparate	500	RF	0.784	0.782	0.775	0.936	1225
VCU	prostate	BonesSeparate	500	LR	0.781	0.74	0.75	0.928	1225
VCU	prostate	BonesSeparate	500	SVM_Linear	0.775	0.764	0.76	0.929	1225
VCU	prostate	BonesSeparate	500	SVM_RBF	0.773	0.766	0.762	0.931	1225
VCU	prostate	BonesSeparate	1000	RF	0.78	0.775	0.769	0.933	1225
VCU	prostate	BonesSeparate	1000	LR	0.781	0.736	0.749	0.93	1225
VCU	prostate	BonesSeparate	1000	SVM_Linear	0.775	0.763	0.758	0.93	1225
VCU	prostate	BonesSeparate	1000	SVM_RBF	0.773	0.753	0.752	0.927	1225
VCU	prostate	NoBones	50	RF	0.782	0.751	0.756	0.93	1225
VCU	prostate	NoBones	50	LR	0.779	0.767	0.766	0.936	1225
VCU	prostate	NoBones	50	SVM_Linear	0.778	0.792	0.781	0.94	1225
VCU	prostate	NoBones	50	SVM_RBF	0.786	0.78	0.778	0.94	1225
VCU	prostate	NoBones	100	RF	0.782	0.751	0.758	0.931	1225
VCU	prostate	NoBones	100	LR	0.78	0.775	0.772	0.938	1225
VCU	prostate	NoBones	100	SVM_Linear	0.777	0.789	0.78	0.94	1225
VCU	prostate	NoBones	100	SVM_RBF	0.785	0.787	0.782	0.94	1225
VCU	prostate	NoBones	250	RF	0.784	0.756	0.76	0.932	1225
VCU	prostate	NoBones	250	LR	0.771	0.752	0.75	0.928	1225
VCU	prostate	NoBones	250	SVM_Linear	0.776	0.774	0.769	0.935	1225
VCU	prostate	NoBones	250	SVM_RBF	0.778	0.779	0.774	0.937	1225
VCU	prostate	NoBones	500	RF	0.782	0.756	0.76	0.932	1225
VCU	prostate	NoBones	500	LR	0.771	0.742	0.743	0.925	1225
VCU	prostate	NoBones	500	SVM_Linear	0.77	0.771	0.764	0.931	1225
VCU	prostate	NoBones	500	SVM_RBF	0.777	0.777	0.772	0.936	1225
VCU	prostate	NoBones	1000	RF	0.78	0.769	0.768	0.938	1225
VCU	prostate	NoBones	1000	LR	0.774	0.737	0.741	0.925	1225
VCU	prostate	NoBones	1000	SVM_Linear	0.775	0.778	0.77	0.934	1225
VCU	prostate	NoBones	1000	SVM_RBF	0.777	0.764	0.763	0.932	1225

Table 9. Late Integration Results - VA-ROQS dataset Lung Disease specific macro-averaged Precision, Recall, F_1-Score, and Overall Accuracy.

Disease	Image Features Type	Image Feature Size	Integration Type	Precision	Recall	F_1-Score	Acc	Support
lung	BonesSeparate	50	AVG	0.918	0.824	0.857	0.946	2564
lung	BonesSeparate	50	MAX	0.916	0.825	0.858	0.946	2564
lung	BonesSeparate	100	AVG	0.918	0.84	0.868	0.946	2564
lung	BonesSeparate	100	MAX	0.916	0.84	0.867	0.945	2564
lung	BonesSeparate	250	AVG	0.924	0.84	0.869	0.947	2564
lung	BonesSeparate	250	MAX	0.921	0.842	0.87	0.947	2564
lung	BonesSeparate	500	AVG	0.918	0.811	0.849	0.945	2564
lung	BonesSeparate	500	MAX	0.919	0.821	0.856	0.945	2564
lung	BonesSeparate	1000	AVG	0.923	0.797	0.839	0.943	2564
lung	BonesSeparate	1000	MAX	0.924	0.811	0.852	0.944	2564
lung	NoBones	50	AVG	0.93	0.842	0.874	0.947	2564
lung	NoBones	50	MAX	0.932	0.86	0.884	0.948	2564
lung	NoBones	100	AVG	0.933	0.859	0.884	0.948	2564
lung	NoBones	100	MAX	0.927	0.859	0.882	0.947	2564
lung	NoBones	250	AVG	0.929	0.805	0.848	0.946	2564
lung	NoBones	250	MAX	0.928	0.822	0.861	0.946	2564
lung	NoBones	500	AVG	0.924	0.822	0.86	0.945	2564
lung	NoBones	500	MAX	0.928	0.843	0.875	0.947	2564
lung	NoBones	1000	AVG	0.929	0.812	0.853	0.945	2564
lung	NoBones	1000	MAX	0.927	0.817	0.857	0.946	2564

Table 10. Late Integration Results - VA-ROQS dataset Prostate Disease specific macro-averaged Precision, Recall, F_1-Score, and Overall Accuracy.

Disease	Image Features Type	Image Feature Size	Integration Type	Precision	Recall	F_1-Score	Acc	Support
prostate	BonesSeparate	50	AVG	0.896	0.836	0.856	0.929	4020
prostate	BonesSeparate	50	MAX	0.898	0.85	0.866	0.932	4020
prostate	BonesSeparate	100	AVG	0.897	0.836	0.857	0.93	4020
prostate	BonesSeparate	100	MAX	0.897	0.848	0.864	0.93	4020
prostate	BonesSeparate	250	AVG	0.898	0.829	0.851	0.926	4020
prostate	BonesSeparate	250	MAX	0.9	0.841	0.86	0.928	4020
prostate	BonesSeparate	500	AVG	0.904	0.819	0.847	0.926	4020
prostate	BonesSeparate	500	MAX	0.9	0.831	0.853	0.926	4020
prostate	BonesSeparate	1000	AVG	0.9	0.803	0.833	0.922	4020
prostate	BonesSeparate	1000	MAX	0.899	0.829	0.852	0.926	4020
prostate	NoBones	50	AVG	0.903	0.825	0.851	0.929	4020
prostate	NoBones	50	MAX	0.906	0.829	0.855	0.929	4020
prostate	NoBones	100	AVG	0.906	0.818	0.848	0.928	4020
prostate	NoBones	100	MAX	0.905	0.826	0.853	0.929	4020
prostate	NoBones	250	AVG	0.907	0.821	0.85	0.928	4020
prostate	NoBones	250	MAX	0.904	0.827	0.853	0.929	4020
prostate	NoBones	500	AVG	0.908	0.819	0.849	0.927	4020
prostate	NoBones	500	MAX	0.904	0.826	0.853	0.928	4020
prostate	NoBones	1000	AVG	0.91	0.813	0.846	0.927	4020
prostate	NoBones	1000	MAX	0.897	0.824	0.848	0.925	4020

Table 11. Late Integration Results - VCU dataset Prostate - Disease specific macro-averaged Precision, Recall, F_1-Score, and Overall Accuracy.

Disease	Image Features Type	Image Feature Size	Integration Type	Precision	Recall	F_1-Score	Acc	Support
prostate	BonesSeparate	50	AVG	0.799	0.688	0.719	0.927	1225
prostate	BonesSeparate	50	MAX	0.8	0.713	0.738	0.935	1225
prostate	BonesSeparate	100	AVG	0.802	0.685	0.719	0.929	1225
prostate	BonesSeparate	100	MAX	0.801	0.708	0.739	0.936	1225
prostate	BonesSeparate	250	AVG	0.804	0.672	0.705	0.927	1225
prostate	BonesSeparate	250	MAX	0.799	0.703	0.731	0.932	1225
prostate	BonesSeparate	500	AVG	0.802	0.673	0.709	0.928	1225
prostate	BonesSeparate	500	MAX	0.795	0.703	0.733	0.935	1225
prostate	BonesSeparate	1000	AVG	0.805	0.675	0.71	0.931	1225
prostate	BonesSeparate	1000	MAX	0.807	0.715	0.746	0.94	1225
prostate	NoBones	50	AVG	0.786	0.657	0.7	0.92	1225
prostate	NoBones	50	MAX	0.792	0.707	0.734	0.933	1225
prostate	NoBones	100	AVG	0.791	0.68	0.713	0.922	1225
prostate	NoBones	100	MAX	0.791	0.711	0.734	0.931	1225
prostate	NoBones	250	AVG	0.794	0.683	0.716	0.925	1225
prostate	NoBones	250	MAX	0.793	0.718	0.741	0.933	1225
prostate	NoBones	500	AVG	0.795	0.683	0.718	0.927	1225
prostate	NoBones	500	MAX	0.791	0.713	0.737	0.932	1225
prostate	NoBones	1000	AVG	0.803	0.684	0.725	0.932	1225
prostate	NoBones	1000	MAX	0.792	0.704	0.734	0.931	1225

Table 12. Late Integration Results - VCU dataset Lung - Disease specific macro-averaged Precision, Recall, F_1-Score, and Overall Accuracy.

Disease	Image Features Type	Image Feature Size	Integration Type	Precision	Recall	F_1-Score	Acc	Support
lung	BonesSeparate	50	AVG	0.858	0.807	0.811	0.964	955
lung	BonesSeparate	50	MAX	0.853	0.812	0.810	0.964	955
lung	BonesSeparate	100	AVG	0.858	0.807	0.811	0.964	955
lung	BonesSeparate	100	MAX	0.849	0.81	0.806	0.963	955
lung	BonesSeparate	250	AVG	0.867	0.812	0.822	0.966	955
lung	BonesSeparate	250	MAX	0.867	0.812	0.822	0.966	955
lung	BonesSeparate	500	AVG	0.879	0.835	0.827	0.963	955
lung	BonesSeparate	500	MAX	0.883	0.854	0.846	0.968	955
lung	BonesSeparate	1000	AVG	0.866	0.804	0.815	0.964	955
lung	BonesSeparate	1000	MAX	0.885	0.868	0.859	0.971	955
lung	NoBones	50	AVG	0.866	0.811	0.819	0.966	955
lung	NoBones	50	MAX	0.867	0.816	0.824	0.968	955
lung	NoBones	100	AVG	0.877	0.816	0.826	0.969	955
lung	NoBones	100	MAX	0.893	0.858	0.85	0.97	955
lung	NoBones	250	AVG	0.881	0.844	0.837	0.965	955
lung	NoBones	250	MAX	0.882	0.853	0.843	0.968	955
lung	NoBones	500	AVG	0.876	0.797	0.806	0.964	955
lung	NoBones	500	MAX	0.873	0.848	0.831	0.965	955
lung	NoBones	1000	AVG	0.875	0.798	0.815	0.964	955
lung	NoBones	1000	MAX	0.877	0.808	0.825	0.966	955

Table 13. Single-View Label wise results - VA-ROQS dataset Lung, BonesSeparate, with 100 Image Features

Structure Name	Precision	Recall	F-Score	Support
BrachialPlexus	0.78	0.37	0.50	19
Esophagus	0.92	0.87	0.89	126
Heart	0.87	0.99	0.92	134
Other	0.92	0.98	0.95	1998
PTV	0.50	0.01	0.03	143
SpinalCord	0.97	0.95	0.96	144
accuracy	0.92	0.92	0.92	2564
macro avg	0.83	0.69	0.71	2564
weighted avg	0.89	0.92	0.89	2564

Table 14. Single-View Label wise results - VA-ROQS dataset Prostate, BonesSeparate, with 100 Image Features

Structure Name	Precision	Recall	F-Score	Support
Bladder	0.60	0.65	0.62	146
Femur_L	0.97	0.92	0.95	190
Femur_R	0.98	0.93	0.95	191
LargeBowel	0.72	0.27	0.39	105
Other	0.86	0.96	0.91	2954
PTV	0.58	0.08	0.13	185
Rectum	0.78	0.61	0.69	189
SmallBowel	0.57	0.22	0.31	60
accuracy	0.86	0.86	0.86	4020
macro avg	0.76	0.58	0.62	4020
weighted avg	0.84	0.86	0.83	4020

Table 15. Single-View Label wise results - VCU dataset Lung, BonesSeparate, with 100 Image Features

Structure Name	Precision	Recall	F-Score	Support
BrachialPlexus	0.00	0.00	0.00	4
Esophagus	0.82	0.66	0.73	47
Heart	0.98	0.91	0.94	45
Other	0.92	0.99	0.95	775
PTV	0.00	0.00	0.00	36
SpinalCord	0.95	0.83	0.89	48
accuracy	0.92	0.92	0.92	955
macro avg	0.61	0.57	0.59	955
weighted avg	0.88	0.92	0.90	955

Table 16. Single-View Label wise results - VCU dataset Prostate, BonesSeparate, with 100 Image Features

Structure Name	Precision	Recall	F-Score	Support
Bladder	0.83	0.30	0.44	50
Femur_L	0.96	0.90	0.93	29
Femur_R	0.93	0.90	0.91	29
LargeBowel	0.00	0.00	0.00	0
Other	0.87	0.98	0.92	980
PTV	0.20	0.03	0.05	38
Rectum	0.88	0.60	0.71	50
SmallBowel	1.00	0.10	0.19	49
accuracy	0.87	0.87	0.87	1225
macro avg	0.71	0.48	0.52	1225
weighted avg	0.86	0.87	0.84	1225

Table 17. Intermediate Integration Label wise results - VA-ROQS dataset Lung, BonesSeparate, 50 Image Features, 200 Text Features, with Random Forest Algorithm

Structure Name	Precision	Recall	F-Score	Support
BrachialPlexus	1.00	0.16	0.27	19
Esophagus	0.95	1.00	0.97	126
Heart	0.97	1.00	0.99	134
Other	0.94	0.99	0.96	1998
PTV	0.79	0.41	0.54	143
SpinalCord	0.98	0.90	0.93	144
accuracy	0.94	0.94	0.94	2564
macro avg	0.94	0.74	0.78	2564
weighted avg	0.94	0.94	0.94	2564

Table 18. Intermediate Integration Label wise results - VCU dataset Lung, BonesSeparate,, 50 Image Features, 200 Text Features, with Random Forest Algorithm

Structure Name	Precision	Recall	F-Score	Support
BrachialPlexus	0.50	0.50	0.50	4
Esophagus	1.00	1.00	1.00	47
Heart	1.00	1.00	1.00	45
Other	0.99	0.97	0.98	775
PTV	0.89	0.92	0.90	36
SpinalCord	0.75	0.98	0.85	48
accuracy	0.97	0.97	0.97	955
macro avg	0.86	0.89	0.87	955
weighted avg	0.97	0.97	0.97	955

Table 19. Intermediate Integration Label wise results - VA-ROQS dataset Prostate, BonesSeparate, 50 Image Features, 200 Text Features, with Random Forest Algorithm

Structure Name	Precision	Recall	F-Score	Support
Bladder	0.75	1.00	0.86	146
Femur_L	0.86	0.98	0.92	190
Femur_R	0.86	0.99	0.92	191
LargeBowel	0.96	0.83	0.89	105
Other	0.97	0.94	0.95	2954
PTV	0.78	0.58	0.66	185
Rectum	0.98	0.97	0.97	189
SmallBowel	0.62	0.88	0.73	60
accuracy	0.93	0.93	0.93	4020
macro avg	0.85	0.90	0.86	4020
weighted avg	0.93	0.93	0.93	4020

Table 20. Intermediate Integration Label wise results - VCU dataset Prostate, BonesSeparate, 50 Image Features, 200 Text Features, with Random Forest Algorithm

Structure Name	Precision	Recall	F-Score	Support
Bladder	1.00	1.00	1.00	50
Femur_L	0.94	1.00	0.97	29
Femur_R	0.97	1.00	0.98	29
LargeBowel	0.00	0.00	0.00	0
Other	0.97	0.95	0.96	980
PTV	0.34	0.53	0.42	38
Rectum	1.00	1.00	1.00	50
SmallBowel	1.00	0.86	0.92	49
accuracy	0.94	0.94	0.94	1225
macro avg	0.78	0.79	0.78	1225
weighted avg	0.96	0.94	0.95	1225

Table 21. Late Integration Label wise results - VA-ROQS dataset Lung, 100 Image Features, 200 Text Features

Structure Name	Precision	Recall	F-Score	Support
BrachialPlexus	0.88	0.79	0.83	19
Esophagus	0.97	0.98	0 .98	126
Heart	0.96	1.00	0.98	134
Other	0.95	0.98	0.97	1998
PTV	0.76	0.38	0.51	143
SpinalCord	0.98	0.90	0.93	144
accuracy	0.95	0.95	0.95	2564
macro avg	0.92	0.84	0.87	2564
weighted avg	0.94	0.95	0.94	2564

Table 22. Late Integration Label wise results - VCU dataset Lung, BonesSeparate, 100 Image Features, 200 Text Features

Structure Name	Precision	Recall	F-Score	Support
BrachialPlexus	0.25	0.50	0.33	4
Esophagus	1.00	0.98	0.99	47
Heart	1.00	1.00	1.00	45
Other	0.97	0.99	0.98	775
PTV	0.94	0.42	0.58	36
SpinalCord	0.94	0.98	0.96	48
accuracy	0.96	0.96	0.96	955
macro avg	0.85	0.81	0.81	955
weighted avg	0.97	0.96	0.96	955

Table 23. Late Integration Label wise results - VA-ROQS dataset Prostate, BonesSeparate, 100 Image Features, 200 Text Features

Structure Name	Precision	Recall	F-Score	Support
Bladder	0.76	1.00	0.86	146
Femur_L	0.90	0.92	0.91	190
Femur_R	0.92	0.92	0.92	191
LargeBowel	0.97	0.83	0.89	105
Other	0.95	0.96	0.95	2954
PTV	0.80	0.51	0.62	185
Rectum	0.98	0.96	0.97	189
SmallBowel	0.91	0.68	0.78	60
accuracy	0.96	0.93	0.93	4020
macro avg	0.90	0.85	0.86	4020
weighted avg	0.93	0.93	0.93	4020

Table 24. Late Integration Label wise results - VCU dataset Prostate, BonesSeparate, 100 Image Features, 200 Text Features

Structure Name	Precision	Recall	F-Score	Support
Bladder	1.00	0.92	0.96	50
Femur_L	0.97	0.97	0.97	29
Femur_R	1.00	0.97	0.98	29
LargeBowel	0.00	0.00	0.00	0
Other	0.94	0.98	0.96	980
PTV	0.50	0.45	0.47	38
Rectum	1.00	0.98	0.99	50
SmallBowel	1.00	0.41	0.58	49
accuracy	0.94	0.94	0.94	1225
macro avg	0.80	0.71	0.74	1225
weighted avg	0.94	0.94	0.93	1225
